# Real-time PCR assays for diagnosing brucellar spondylitis using formalin-fixed paraffin-embedded tissues

**DOI:** 10.1097/MD.0000000000010062

**Published:** 2018-03-02

**Authors:** Man Li, Xingang Zhou, Jingjing Li, Lei Sun, Xiangmei Chen, Peng Wang

**Affiliations:** a Department of Pathology; b Department of Radiology, Beijing Ditan Hospital, Capital Medical University, Chaoyang District, Beijing, China.

**Keywords:** *B melitensis*, brucellar spondylitis, real-time PCR, standard tube agglutination test

## Abstract

It is difficult to diagnose brucellar spondylitis because of its nonspecific clinical, radiological, and histological characteristics. This study aimed to determine whether real-time polymerase chain reaction (PCR) using formalin-fixed paraffin-embedded (FFPE) tissues was superior to conventional serum-based methods for diagnosing brucellar spondylitis.

This retrospective study included 31 patients with brucellosis and a control group of 20 people with no history of brucellosis or exposure to *Brucella* spp. Samples from all patients with brucellar spondylitis were evaluated using Giemsa staining, the standard tube agglutination (STA) test, blood culture, and real-time PCR.

The brucellar spondylitis was acute in 7 patients (22.6%), subacute in 15 patients (48.4%), and chronic in 9 patients (29%). Serological assays provided positive results for 25 patients (80.1%), real-time PCR provided positive results for 29 patients (93.5%), and blood cultures provided positive results for 11 patients (35.5%). The real-time PCR provided sensitivity of 93.5%, specificity of 100%, a positive predictive value of 100%, and a negative predictive value of 100%. The corresponding values for the STA test were 80.1%, 100%, 100%, and 76.9%, respectively. Real-time PCR provided better sensitivity than Giemsa staining, the STA test, and blood culture, although the difference between PCR and STA was not statistically significant (*P* = .22). *B melitensis* was the only pathogen that was detected in patient with brucellar spondylitis using real-time PCR.

These results suggest that real-time PCR provides a high sensitivity for diagnosing brucellar spondylitis. Furthermore, the real-time PCR results indicate that *B melitensis* was the causative pathogen in these cases.

## 1 Introduction

Brucellosis is a form of zoonosis caused by *Brucella* spp and is endemic in various parts of the world, especially in Mediterranean, Asia, Africa, and South America regions.^[1,2]^*Brucella* spp are intracellular bacterial pathogens that are capable of infecting animals and humans. The *Brucella* genus consists of 6 classic species, with *B melitensis*, *B abortus*, *B suis*, and *B canis* being considered pathogenic for humans, while *B ceti* and *B pinnipedialis* are isolated from sea mammals^[3,4]^ and occasionally cause disease in humans.^[5]^ Transmission of brucellosis from animals to humans mainly involves direct contact with infected animals, ingestion of raw dairy products, or consumption of infected meat from domestic livestock.^[6]^ Human brucellosis has various clinical manifestations, including fever, sweating, chills, headache, and arthralgia of the large joints.^[7]^The most common complications of brucellosis are osteoarticular manifestations, which affect approximately one-third of patients and include sacroiliitis, peripheral arthritis, spondylitis, osteomyelitis, and bursitis. Spondylitis is the most prevalent and important clinical manifestation of osteoarticular involvement in adults who are infected by *Brucella* spp.^[8]^ However, it is difficult to diagnose brucellar spondylodiscitis, as the clinical findings are usually nonspecific and the radiological features can mimic those of other bacterial, fungal, inflammatory, and neoplastic diseases.^[9]^ Conventional laboratory tests are time-consuming, involve risk that is associated with handling live cultures, and require expert interpretation. Thus, methods to achieve species-level identification are needed to decipher this disease's epidemiology, facilitate accurate diagnoses, and implement effective control measures.

Polymerase chain reaction (PCR) assays may be a sensitive and specific technique for detecting and identifying *Brucella* spp in peripheral blood and other tissues.^[10]^ Furthermore, real-time PCR can be used to diagnose human cases of brucellosis and can be combined with clinical findings to differentiate between the inactive, seropositive, and active states.^[11,12]^ Real-time PCR has also recently been used to detect *Brucella* spp, with differentiation at the species level using unique genetic loci for *B melitensis*, *B abortus*, *B suis*, and *B canis*. Therefore, the present study examined whether real-time PCR could be used to diagnose brucellar spondylitis by amplifying *Brucella* genomic signatures from formalin-fixed paraffin-embedded (FFPE) specimens, and whether real-time PCR was superior to conventional diagnostic methods. A real-time PCR assay was developed using 4 specific reactions to rapidly detect and identify *Brucella* spp in human cases of brucellar spondylitis.

## 2 Methods

### 2.1 Patients

This study involved 31 patients (11 women and 20 men; mean age: 54 years, range: 33–69 years) who were diagnosed with brucellar spondylitis and hospitalized at Beijing Ditan Hospital, Capital Medical University, China, between January 2013 and January 2017. The patients’ histories were obtained and physical examinations and routine laboratory tests were performed. The diagnosis of brucellosis was established based on isolation of *Brucella* spp from a blood culture and/or the presence of compatible clinical findings plus a standard tube agglutination (STA) test result of ≥1:160 or a Coombs anti-*Brucella* test titer of ≥1:320.^[13]^ The diagnosis of brucellar spondylitis was supported by infection in at least 1 vertebra or intervertebral disc (based on magnetic resonance imaging [MRI]) plus positive clinical findings. Surgery was performed for all patients with spinal instability or radiculopathy.

### 2.2 Histochemistry

Biopsy tissues were fixed in freshly prepared neutral formalin solution and processed using FFPE procedures. Tissue sections (5 μm) were mounted on positively charged glass slides, and serial sections were stained using hematoxylin and eosin. The slides were also stained using Brown–Hopps tissue Gram stain and a 3% Giemsa solution. Fixed slides were also immersed in ZN Carbol Fuchsin stain for 20 to 25 minutes (desiccation was avoided by adding more stain when needed), rinsed under slow running tap water, and air dried.

### 2.3 Magnetic resonance imaging

All MRI scans were performed using a 1.5-T unit with gradient echoplanar capabilities and a standard phased-array surface receiver coil for imaging the spine. The following spinal MRI sequences were selected: axial and sagittal spin-echo T1-weighted images (TR/TE, 520/14), axial and sagittal turbo spin-echo T2-weighted images (4000/99), sagittal fat-suppressed T2-weighted images, and contrast-enhanced axial and sagittal spin-echo T1-weighted images (0.1 mmol/kg of intravenous gadopentetate dimeglumine [Magnevist, Schering]). The MRI parameters were: fields of view of 20 to 25 cm for the axial plane and 30 to 35 cm for the sagittal plane, 2 excitations, a matrix size of 256 × 132, slice thickness of 4 mm, an intersection gap of 1 mm, and an echo-train length of 8. The signal changes and enhancement at the intervertebral discs, vertebral bodies, vertebral endplates, facet joints, paravertebral soft tissues, and epidural spaces were assessed by 2 radiologists before a diagnosis was made.

### 2.4 Bacteriological culture

Blood cultures were performed using a semiautomatic BACTEC 9240 system (Becton Dickinson Diagnostic Instrument Systems, NJ). If no growth was detected during the standard 5-day period, the incubation was maintained for 15 days and blind subcultures were plated on *Brucella* agar (Becton Dickinson) after 7 and 15 days. The isolates were identified based on their requirement for CO_2_, H_2_S production, urease and oxidase positivity, growth in thionine, and positive agglutination with specific antiserum.

### 2.5 Serological testing

Plain antigen from *B abortus* was used for the STA, and serial dilution of the sera was performed using a phosphate-buffered saline solution (1:10 to 1:1280). A 0.5-mL aliquot of a 10% solution of *B abortus* was added to each tube, which was subsequently incubated at 37 °C for 24 hours. All tubes were compared to control tubes that lacked the antigen to examine their opacity. Sera with positive results at titers of ≥1:160 were considered clinically positive.

### 2.6 DNA extraction

Five sections of FFPE tissues were collected into DNase-free tubes for nucleic acid extraction, which was performed using an FFPE DNA extraction kit (QIAGEN, Valencia, CA). The extraction protocol involved deparaffinization using xylene and ethanol washes, tissue digestion using incubation with Proteinase K (20 mg/mL) at 50 °C for ≥1 hour (depending on the size of the sections), RNA removal using incubation with RNase A (100 mg/mL) at 37 °C for 30 minutes, DNA recovery using washes and column elutions (30 mL elution volume for each block), and quantification of the DNA samples (NanoDrop2000; Thermo Fisher Scientific, Wilmington, DE). Total DNA samples were stored at −20 °C for later testing.

### 2.7 Real-time PCR

Regions within the following open reading frames were chosen to develop primers for species differentiation: BMEII0466 for *B melitensis*, BruAb2_0168 for *B abortus*, BR0952 for *B suis*, and BMEII0635-0636 for *B canis*. The primer pairs were: *B melitensis*, forward: 5′-TCGCATCGGCAGTTTCAA-3′ and reverse: 5′-CCAGCTTTTGGCCTTTTCC-3′; *B abortus*, forward: 5′-GCACACTCACCTTCCACAACAA-3′ and reverse: 5′-CCCCGTTCTGCACCAGACT-3′; *B suis*, forward: 5′-CCTGCAAAAAGCAGCAACCA-3′ and reverse: 5′-CCTCCGCCAGTCGTGAAA-3′; and *B canis*, forward: 5′-AAAATGCGGATCGGCCTT-3′ and reverse: 5′-TCCCGGCGCATTGCT-3′. The real-time PCR was performed using a Roche Z480 system according to the manufacturer's protocol. The PCR mixtures were subjected to an initial denaturation at 95 °C for 3 minutes, which was followed by 35 cycles of amplification (denaturation at 95 °C for 30 seconds, annealing at 60 °C for 30 seconds, and elongation at 72 °C for 20 seconds), and then final elongation at 72 °C for 10 minutes. The resulting PCR products were separated using agarose gel, purified for cloning, and sent to an external supplier for processing (Genewiz, Tianjin, China).

### 2.8 Statistical analysis

Data were analyzed using IBM SPSS software (version 19.0; IBM Corp, Armonk, NY). Differences between brucellosis cases in the acute (<3 months), subacute (3–12 months), and chronic (>12 months) states were evaluated using Student independent *t* test. Categorical variables were analyzed using the χ^2^ test. Observed agreement and Kappa values were calculated to assess the reliability of the four diagnostic methods. In addition, we calculated sensitivity, specificity, positive predictive, and negative predictive values for each diagnostic method. Differences were considered significant at *P*-values of <.05.

## 3 Results

### 3.1 Patient characteristics

Seven patients (22.6%) had acute disease, 15 patients (48.4%) had subacute disease, and 9 patients (29%) had chronic disease. Nineteen patients (61.8%) had a history of raising livestock, another 5 patients (16.1%) had other occupations with a high risk of brucellosis (3 veterinarians and 2 butchers), and 7 patients (22.6%) had no occupational risk of brucellosis. The patients’ clinical symptoms varied according to disease stage, with fever and appetite loss being more common in the acute stage than in the subacute stage (*P* = .068) or chronic stage (*P* = .107). No stage-specific differences were observed in terms of sweating, headache, chills, cough, weight loss, back pain, chest pain, joint pain, or muscle aches.

### 3.2 Histopathological findings

**Figure 1:**
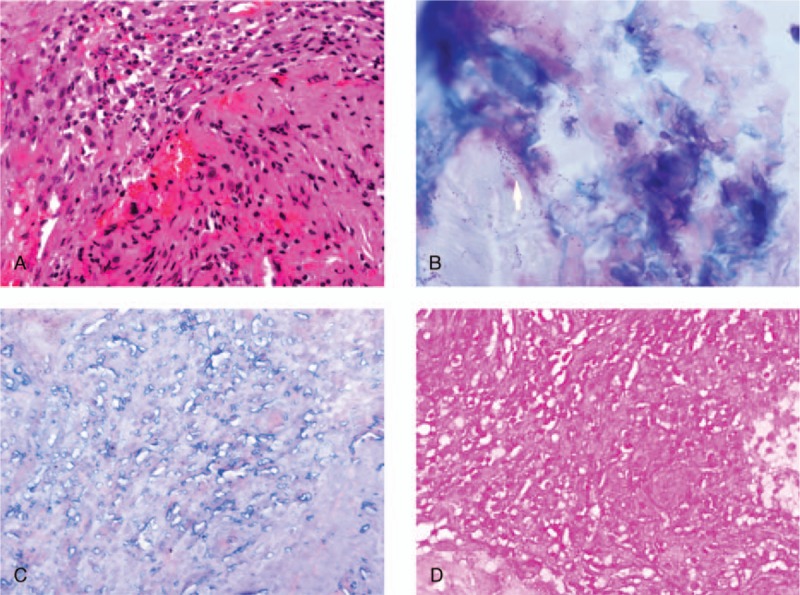
Nucleus pulposus biopsy results. (A) Hematoxylin and eosin staining (×400). (B) Microscopic examination reveals bacilli (Giemsa stain, ×1000). (C) Ziehl-Neelsen stain (×400). (D) Gram-negative *Brucella* spp (Gram staining, ×400).

Nucleus pulposus biopsy was performed and revealed abundant inflammatory cell infiltration. The walls of the small blood vessels were infiltrated by mixed inflammatory cells (Fig. 1A). Giemsa staining revealed large numbers of bacilli (Fig. 1B), although negative results were observed for acid-fast staining (Fig. 1C). Gram staining revealed gram-negative *Brucella* in the lumbar vertebrae (Fig. 1D).

### 3.3 MRI findings

**Figure 2:**
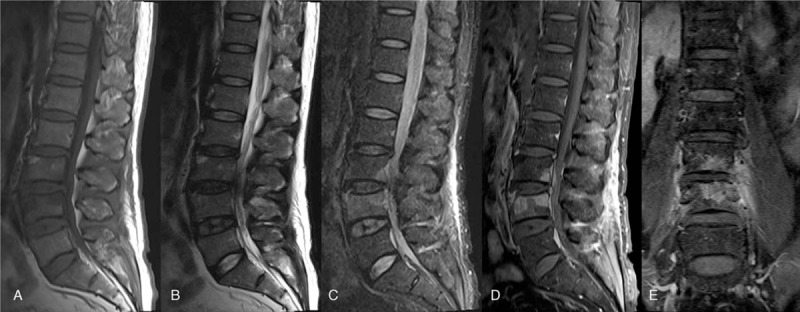
Magnetic resonance imaging findings. (A) A sagittal spin-echo T1-weighted image reveals decreased signal intensity in the bodies of the L3/4 vertebrae. (B) A T2-weighted image reveals increased signal intensity involving the L3/4 disk. (C) A fat-suppressed T2-weighted image reveals hyperintense signal intensities corresponding to the same level. (D) A sagittal gadolinium-enhanced T1-weighted image reveals areas of enhancement in the affected vertebral bodies and disks. (E) A coronal image reveals abnormally strong signals in L3/4.

Lesions were presented in the vertebral body marrow with abnormal signal intensity and compression of cord and root. No collapsed vertebral body or angulation deformity, including kyphosis and scoliosis were observed in patients. For the 31 enrolled patients with spinal brucellosis, L4/5 were involved in 12 cases, L3/S4 were involved in 8 cases, L5/S1 were involved in 4 cases, L2/3 were involved in 4 cases, L1/2 were involved in 2 cases, and T12/L1 were involved in 1 case. Additionally, there were 5 cases who had over 2 regions of vertebral lesions. Low signal intensities were revealed by the T1-weighted images, whereas high signal intensities were revealed by T2-weighted images, including vertebral bodies, endplates, and intervertebral disc spaces. Moreover, remarkable enhancement of affected vertebrae and discs during acute stage of spinal brucellosis were observed in the contrast-enhanced T1-weighted images. However, there were a little or no heterogeneous enhancement of affected vertebrae and discs were observed in the subacute and chronic stages. Radiological examinations from 1 representative patient with spinal brucellosis were shown in Fig. 2.

### 3.4 Real-time PCR results

Positive real-time PCR results were detected for 29 of the 31 patients (93.5%). The negative results were observed for 1 patient in the acute group (sensitivity: 85.7%) and 1 patient in the subacute group (sensitivity: 90.1%). Both patients in the chronic group had positive real-time PCR results. The real-time PCR assay provided overall sensitivity of 93.5%, specificity of 100%, a positive predictive value of 100%, and a negative predictive value of 90.9%. None of the control samples provided positive results for *B melitensis*, *B abortus*, *B suis*, or *B canis*. All of the positive results involved amplification of the *B melitensis* marker.

### 3.5 Results from the other assays

The STA test provided positive results for 25 patients, which corresponded to sensitivity of 80.6%, specificity of 100%, a positive predictive value of 100%, and a negative predictive value of 76.9%. Blood cultures revealed the presence of *Brucella* spp in only 11 patients (35.5%), and all positive results were obtained within 7 days of incubation. The sensitivity, specificity, positive predictive, and negative predictive values for Giemsa staining, the STA test, blood culture, and real-time PCR are shown in Table 1. The agreement between the findings for real-time PCR, Giemsa staining, the STA test, and blood culture are shown in Table 2. Real-time PCR was significantly more sensitive than Giemsa staining (*P* = .001) and blood culture (*P* = .021), but was only slightly more sensitive than the STA test (*P* = 0.219). Real-time PCR provided better negative predictive value than the other methods.

**Table 1:**
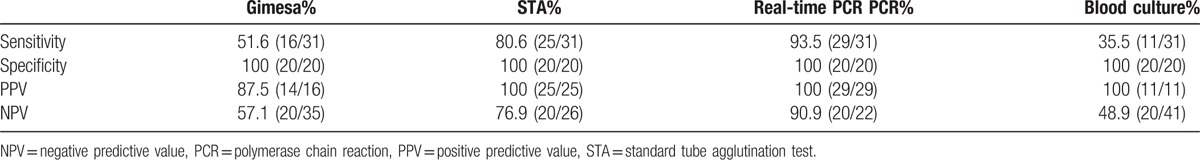
Sensitivity, specificity, positive, and negative predictive values between the 4 methods.

**Table 2:**
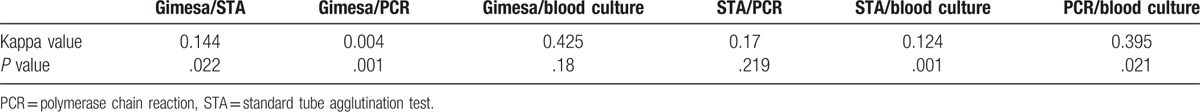
Reliability of the 4 methods for the diagnosis of brucellosis spondylitis.

## 4 Discussion

Brucellosis is one of the serious zoonosis that is distributed worldwide. The high morbidity of brucellosis poses great challenge in many countries.^[14]^*Brucella* spp, a facultative intracellular micro-organism, is strong enough to survive and multiply in cells of mononuclear phagocyte system, which leads to the strong tendency of brucellosis. The abovementioned characteristics of *Brucella* spp lead to the long-term musculoskeletal symptoms, focal complications, and relapse of brucellosis.^[15,16]^ Complications of brucellosis are various, including peripheral arthritis, sacroiliitis, and spondylodiscitis-like joint disorders.^[17]^ For older patients infected with brucellosis, spondylodiscitis is frequently involved in, especially for those older than 50 years. So far, the diagnosis of brucellar spondylitis needs to be considered in many examinations, including computed tomography, MRI, and bone scintigraphy.^[18]^ Unfortunately, spinal brucellosis and other spinal diseases are difficult to distinguish, including pyogenic osteomyelitis and tuberculous spondylitis, which present similar imaging features with spinal brucellosis.^[19]^ Diagnosis of spinal brucellosis cannot simply rely on the conventional clinical and laboratory examinations.

The gold standard for diagnosing brucellosis is isolation of *Brucella* spp, although this process is time consuming and associated with a risk of laboratory-acquired infections. In addition, culture sampling often has low sensitivity, which is dependent on the culture medium, specific *Brucella* species, disease stage, and quantity of circulating bacteria.^[20]^ Serological tests seem to be more effective that blood cultures, but the results can be unspecific because of cross-reaction or subsensitive reactions in samples from areas with a low or subclinical prevalence of brucellosis.^[21]^ Moreover, it is difficult to serologically distinguish between *Brucella* spp. Histopathological results are also difficult to interpret, as the findings can resemble *Burkholderia pseudomallei* or *Mycobacterium tuberculosis*, which cause melioidosis and tuberculosis.^[22]^ Real-time PCR may help address these issues, and technological advances have helped improve its sensitivity, specificity, and technical challenges, as well as lower its cost.^[23]^ Thus, a combination of histopathology and real-time PCR have been applied during the bacterial differentiation process.

The present study aimed to confirm the involvement of brucellar spondylitis using real-time PCR techniques to amplify *Brucella* genomic signatures from FFPE specimens. This approach allowed us to identify and differentiate between *B abortus*, *B melitensis*, and *B suis*, in a single <90-minute test. Further analysis of the results revealed that *B melitensis* was the causative pathogen in all cases with positive real-time PCR results, which was confirmed using sequencing analysis. In this context, *B melitensis* is known to be the most important zoonotic species, followed by *B abortus*, *B suis*, and *B canis*. However, human cases of brucellosis can be directly related to the status of animal brucellosis in a particular geographical region. Therefore, in addition to diagnosing brucellosis, it is valuable to identify the causative species, which can help improve our epidemiological knowledge, treatments, and control measures.

The present study revealed that real-time PCR had better sensitivity (93.5%) than blood culture (35.5%), the STA test (80.6%), and Giemsa staining (51.6%). Thus, real-time PCR appears to be a highly specific technique for identifying and differentiating between the 4 *Brucella* species. Furthermore, real-time PCR may be especially useful for patients who have clinical symptoms and negative serological results, as most cases provided positive results using FFPE biopsy specimens. Only 2 cases had negative results for real-time PCR but positive serological results. Therefore, we recommend using both STA and real-time PCR to diagnose cases that may involve spondylitis.

In conclusion, we used real-time PCR assays and FFPE specimens to diagnose brucellar spondylitis, which provided higher sensitivity than Giemsa staining, the STA test, and blood culture. This simple and easily performed assay provides a highly specific technique for identifying and differentiating between the 4 *Brucella* species. It may also be valuable for testing clinical specimens, which generally have a very low bacterial load.
